# INFLAMMATORY BIOMARKERS ASSOCIATED WITH COGNITIVE FUNCTION AND DEMENTIA

**DOI:** 10.1093/geroni/igac059.2266

**Published:** 2022-12-20

**Authors:** Jiachen Chen, Yuan Fang, Kathryn Lunetta, Joanne Murabito, Margaret Doyle

**Affiliations:** Boston University School of Public Health, Boston, Massachusetts, United States; Boston University School of Public Health, Boston, Massachusetts, United States; Boston University School of Public Health, Boston, Massachusetts, United States; Boston University, Boston, Massachusetts, United States; University of Vermont, Colchester, Vermont, United States

## Abstract

Neuroinflammation is an important contributor to the pathogenesis of dementia, but the role of circulating inflammatory markers is unclear. The aim of the study is to identify circulating inflammatory biomarkers associated with cognitive function in the Framingham Heart Study Offspring cohort. We used OLINK inflammation panel to measure 92 inflammation protein biomarkers using stored plasma samples at Offspring examination 7 (1998-2001) from 879 participants (52% female, mean 61 years range 40 to 88 years, 22% APOE e4 carrier). Cognitive function was assessed with seven tests derived from a neuropsychological (NP) testing battery representing four cognitive domains collected closest to the 7th examination. 68 proteins with at least 50% values above the limit of detection (LOD) were clustered with hierarchical clustering, and data below LOD were replaced with LOD/√2. We identified nine clusters of highly correlated proteins and created cluster composite scores on the first principal component of each. Adjusting for age, sex and education, five of the seven NP tests are nominally associated (p < 0.05) with at least one of the protein clusters, and five of the nine clusters are associated with at least one of the NP tests. The executive function test Trails B–Trails A is nominally associated with three clusters, and the Boston Naming Test 30 items version with two clusters, both sharing one common cluster which includes IFN-γ-inducible chemokines involved in inflammatory conditions. Future analyses will explore individual protein associations with cognitive test performance and incident dementia and will investigate the biological relevance of the identified clusters.

